# BA FOR DEPRESSION AND DIABETES MANAGEMENT: EXAMINED VIA SINGLE-CASE DESIGN

**DOI:** 10.1093/geroni/igac059.1881

**Published:** 2022-12-20

**Authors:** Anna Robertson, Leilani Feliciano

**Affiliations:** University of Colorado, Colorado Springs, Colorado Springs, Colorado, United States; University of Colorado Colorado Springs, Colorado Springs, Colorado, United States

## Abstract

Type 2 Diabetes Mellitus (T2DM) is a pervasive, chronic disease whose prevalence increases with age. The most common treatment options for T2DM include medication, dietary changes, and engagement in physical activity, which require considerable lifestyle changes. Further, individuals with T2DM are highly likely to develop physical and mental health comorbidities that can complicate effective T2DM management. In fact, individuals with T2DM and other comorbidities are frequently excluded from behavioral and other interventions in randomized controlled trials due to their comorbidities. Individualized treatment is necessary to address these comorbid diseases and to effectively navigate lifestyle changes. As part of a larger study evaluating an at-home diabetes management program with patients at a Federally Qualified Health Care Facility, the current study utilized a single-case experimental design approach using changing criterion design to evaluate behavioral activation to treat depression and diabetes in a 68 year-old Hispanic woman with cognitive impairment. Across her intake, five treatment sessions, and two follow-up appointments, we collaborated with her to set and achieve behavioral goals while effectively managing her diabetes. By end of treatment, she reported significant improvements in depression and overall well-being, increased engagement in exercises, increased blood glucose tracking, and rated improvement in her overall efficacy in life that maintained through follow-up. Results revealed the effectiveness of behavioral activation in treating comorbidities in a low-income, diverse individual. Attendees will learn about a promising treatment avenue for older individuals with complex medical and psychiatric conditions and the utility of single case design.

